# The Triple-S Advantage of Endoscopic Management in Gastrointestinal Surgery Complications: Safe, Successful, and Savings-Driven

**DOI:** 10.3390/life14010122

**Published:** 2024-01-15

**Authors:** Francesco Vito Mandarino, Emanuele Sinagra, Alberto Barchi, Silvio Danese

**Affiliations:** 1 Department of Gastroenterology and Gastrointestinal Endoscopy, Scientific Institute San Raffaele, Vita-Salute San Raffaele University, 20132 Milan, Italy; barchi.alberto@hsr.it (A.B.); danese.silvio@hsr.it (S.D.); 2 Gastroenterology and Endoscopy Unit, Fondazione Istituto San Raffaele Giglio, 90015 Cefalù, Italy; emanuelesinagra83@googlemail.com

Despite advances in gastrointestinal (GI) surgery, post-operative complications are not entirely avoidable. Esophagectomy, a major GI surgical intervention, is still associated with post-operative adverse events in 59–63.9% of cases [1,2,3], with a third being classified with a Clavian–Dindo grade greater than IIIB [4], and the post-operative mortality rate ranging between 4 and 11% [1,2,3]. In such scenarios, a redo surgery, which is extremely costly and invasive, exposes patients who are already fragile due to underlying diseases and clinical conditions to a high risk of morbidity and mortality. With the aim of avoiding redo surgery, endoscopy has seen significant progress in recent years and is now considered the treatment of choice for most complications following GI surgery.

For post-esophagectomy anastomotic leaks (ALs), Self-Expandable Metal Stents (SEMSs) have been the preferred endoscopic treatment (ET) for years. SEMSs have shown high success rates, ranging from 62% to 90% [5,6], although their use is associated with some adverse events, particularly migration, which occurs in up to 23% of cases [6]. Endoscopic Vacuum Therapy (EVT) has shown higher success rates in treating post-esophagectomy ALs, ranging between 82% and 95%, with a lower rate of complications [7,8,9,10,11]. Furthermore, it has been proven effective as a rescue therapy [12] and for preventive purposes [13]. In recent years, the VAC stent, a new device combining the benefits of EVT and SEMSs, has demonstrated promising results [14,15,16]. Further prospective studies will be needed to validate its efficacy. Other devices that are used for ET of ALs following GI surgery, including bariatric surgery [17], comprise the over-the-scope clip (OTSC) [18], fibrin glue [19], and the Overstitch system [20].

Endoscopic dilatation (ED) is the primary treatment for benign post-esophagectomy and gastrectomy strictures [21,22]. Robust data have demonstrated that balloon and Savary–Gilliard dilators are equally effective in this setting [23]. The most recent guidelines recommend dilation of the esophageal lumen to >14–15 mm as the target for symptomatic improvement [24,25]. In cases of refractory strictures, defined as those where a luminal diameter of >14 mm is not achieved after five sessions of endoscopic dilation at 2-week intervals, the ET algorithm includes intralesional steroid injection, intralesional mitomycin C injection, incisional therapy, or esophageal stent placement (SEMSs or biodegradable) [26,27].

Delayed gastric emptying (DGE) is a common complication after esophagectomy, occurring in about 15% of patients, and can drastically reduce the quality of life by causing malnutrition. DGE is mainly due to the bilateral disconnection of the vagus nerve and the damage to the celiac plexus during surgery, as well as the development of a different pressure gradient in the gastric tubule, which is brought into the thoracic cavity [28]. Targeting the pylorus to treat DGE has represented a revolution in the management of this complication. Several endoscopic treatments have been developed over time, including botulinum toxin injection [29,30], pylorus balloon dilatation [29], and peroral endoscopic myotomy (G-POEM) [31,32], with symptom improvement achieved in up to 85% of patients after the treatment [29].

Endoscopy is also effective in treating post-operative anastomotic bleeding. Through-the-scope (TTS) clips, OTSC, and injection therapy are the available tools for managing this adverse event [33].

ET of complications following GI surgery is now considered successful, safe, and cost-effective.

The treatment is effective, as reported, for anastomotic leaks, strictures, bleeding and post-surgery motility disorders. The adaptability and versatility of endoscopy facilitate the customization of procedures for individual patients, enhancing the rate of successful outcomes.

Endoscopy, by definition, avoids the risk of physical trauma, unlike a redo surgery. Enhanced precision, enabled by advanced imaging, targeting of affected areas accurately, and reducing the risk of damaging adjacent tissues are considerable advantages of this treatment. Additionally, ET implies a lower anesthetic risk, allowing for moderate or deep sedation instead of general anesthesia in some conditions.

In terms of cost-effectiveness, ET often requires less operating time and resources, which translates to lowering the overall cost. In cases of scheduled treatment, this leads to reduced hospital stays and greater patient satisfaction.

The world of ET of post-GI surgery complications is evolving, and in the future, it could be enriched by translational approaches. Evidence from patients’ microbiota might predict patients who are at risk of anastomotic dehiscence and suggest prophylactic treatments or interventions to modify the bacterial flora [34,35,36,37].

In this Special Issue, we invite submissions of papers analyzing ET of complications after malignant and benign gastrointestinal surgery. The objective is to present further evidence and reinforce the notion of endoscopy as a frontline approach in treating such complications, thereby enhancing patient outcomes.
